# Assessing the Nature of Human Brain‐Derived Extracellular Vesicles on Synaptic Activity Via the Development of an Air‐liquid Microfluidic Platform

**DOI:** 10.1002/advs.202511194

**Published:** 2025-10-05

**Authors:** Corentin Bernou, Yukiko Iwasaki, Willy Lutz, Glaucia Almeida, Emilie Béchard, François Delalande, Magali Rompais, Jules Bouget, Barbara Gorda, Chantal Cazevieille, Yonis Bare, Christine Carapito, Sophie Colomb, Gowrishankar Ganesh, Raphael Gaudin

**Affiliations:** ^1^ Institut de Recherche en Infectiologie de Montpellier (IRIM) Univ Montpellier CNRS 1919 route de Mende Montpellier 34293 France; ^2^ UM‐CNRS Laboratoire D'Informatique de Robotique et de Microelectronique de Montpellier (LIRMM) 161, Rue Ada Montpellier 34095 France; ^3^ Laboratoire de Spectrométrie de Masse Bio‐Organique IPHC, UMR 7178, CNRS‐Université de Strasbourg, ECPM Strasbourg 67200 France; ^4^ Infrastructure Nationale de Protéomique ProFI─UAR2048 Strasbourg 67087 France; ^5^ INM INSERM Univ Montpellier Montpellier 34000 France; ^6^ Equipe de droit pénal et sciences forensiques de Montpellier (EDPFM) Univ Montpellier Département de médecine légale Pôle Urgences Centre Hospitalo‐Universitaire de Montpellier 371 Avenue du Doyen Gaston Giraud Montpellier 34295 France

**Keywords:** exosome, local field potential, machine learning, neural plasticity, proteomics

## Abstract

Brain‐Derived Extracellular Vesicles (BDEVs) have been associated with important roles in functional neuron networks. However, the various models that have been used to study these roles fail to account for all the specificities of the human brain. This study presents a microfluidic platform capable of injecting and/or collecting BDEVs from Organotypic culture of *Post‐mortem* Adult human Brain explants (OPAB) cultured at the air‐liquid interface, while measuring electrical activity in real‐time on 3D‐microelectrode arrays (MEA). The platform design and custom‐made program to control the system allows the automatic collection of BDEVs over days. Mass spectrometry analyses highlight that BDEVs are significantly enriched with synaptic proteins, such as Neural cell adhesion molecule, Syntaxin‐1A, and Synaptopodin, known to regulate synaptic plasticity. Using the MEA‐embedded air‐liquid microfluidic platform, it is shown that BDEVs injection on OPAB induces a significant decrease of local field potential compared to mock conditions, in particular for high frequency oscillations. Finally, a machine learning framework, experimentally validated, revealed that the co‐treatment of OPAB with BDEVs and GW4869, an inhibitor of exosome production, can counteract electrical perturbations induced by BDEVs alone. Together, this work provides innovative methodological developments, that contributed to reveal the diverse biological functions of BDEVs on neural activity.

## Graphical abstract

This seminal study highlights the interwoven and tightly regulated functions of brain‐derived extracellular vesicles. Using microfluidics, proteomics, and AI‐based electric signal analyses of cortical brain explants, this work provides a useful resource dataset revealing the composition of human brain‐derived extracellular vesicles obtained from different isolation methods.

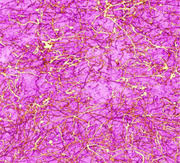

## 1 Introduction

Extracellular vesicles (EVs) are membrane‐bound nanoparticles released by cells that play crucial roles in intercellular communication across various biological systems. EVs refer to vesicles delimited by a lipid bilayer of a wide range of sizes including small (< 200 µm) and large (> 200 µm) EVs. These vesicles can be either of intracellular origin (exosomes) or budding from the plasma membrane (ectosomes).^[^ ^1^ ^]^ Brain‐derived extracellular vesicles (BDEVs) have emerged as major players of the central nervous system (CNS), transporting biochemical and genetic information in an autocrine and paracrine manner.^[^ ^2^ ^]^ BDEVs have been implicated in numerous physiological and pathological processes, including neurodegeneration, brain development, neuronal differentiation and neurogenesis, brain plasticity, synaptic homeostasis, and neurocognitive functions.^[^ ^3^, ^4^, ^5^, ^6^, ^7^ ^]^ The electrophysiological properties of the brain for fast signal transmission across the whole organism relies on the complex arborescence of synaptic structures connecting neurons. Synaptic homeostasis is hence tightly controlled and in this context, BDEVs are emerging as significant regulators of synaptic plasticity.^[^ ^2^, ^5^, ^6^, ^8^ ^]^

BDEVs have been shown to harbor synaptic components, such as AMPA receptors, endocannabinoids, Ephrin receptors, and neurotrophin receptor p75.^[^ ^6^, ^9^, ^10^, ^11^ ^]^ However, the impact of BDEVs on electrical activity is obscure and mostly rely on EVs purified solely from neurons, missing the interwoven network of cell types and the resulting multipartite synapses, as well as the extensive diversity of EVs. Although in vitro 2D and 3D brain cultures surrogates are useful tools, they do not recapitulate the architectural complexity and maturity of an adult human brain. Furthermore, mouse models have been instrumental in our understanding of neurodevelopment and neurocognition, but they can merely be extended to the human brain due to its uniqueness and higher‐order functions.^[^ ^12^ ^]^ Moreover, the species and processing parameters used to study BDEVs are largely influencing the outcome of the investigated process,^[^ ^13^ ^]^ and novel methodological approaches and biosensors are being developed to better characterize BDEV subpopulations and their use as biomarkers.^[^ ^14^ ^]^

The access to well‐preserved *post‐mortem* adult human brain tissues allowed novel insights into the composition of BDEVs in a physiological context and in the context of Alzheimer's disease.^[^ ^15^, ^16^ ^]^ Isolation of BDEVs produced by brain samples requires gentle dissociation of the tissues by proteases, which can result in the loss of proteins harboured at the surface of BDEVs, and increase the risk of contamination of the BDEV preparation by cellular components. The organotypic culture of *post‐mortem* adult human brain explants (OPAB) has been optimized in the past few years to study ex vivo the impact of neurotropic viral infections, and ongoing efforts aim at developing this approach for pre‐clinical drug discovery.^[^ ^17^, ^18^, ^19^, ^20^ ^]^ OPABs exhibit significant spontaneous local field potential activity and synaptic plasticity,^[^ ^17^, ^21^ ^]^ and BDEVs have been shown to modulate the electrical activity of neurons.^[^ ^22^, ^23^ ^]^ Hence, the prolonged culture of adult human brain explants ex vivo provides a great opportunity to evaluate the impact of BDEVs in integrated model systems of physiological relevance.

Here, we designed a novel automated microfluidic platform suitable for OPAB culture at the air‐liquid interface (ALI), allowing for continuous EV collection and supplementation while monitoring the electrophysiological activity of the explant using 3D‐MEAs over up to 72 h. Using this system, we periodically collected OPAB‐derived BDEVs from four donors under static or microfluidic conditions. The BDEVs were isolated using either differential ultracentrifugation (dUC), size exclusion chromatography (SEC), or tissue dissociation for subsequent label‐free mass spectrometry analysis. Besides providing a robust dataset of the global composition of adult human BDEVs, our bioinformatic analyses revealed that BDEVs incorporate a significant proportion of synaptic components, further suggesting a role for BDEV in the modulation of synaptic homeostasis. Electrophysiological monitoring of the spontaneous LFP of OPAB from five donors coupled to machine learning algorithms highlighted that BDEVs significantly dampen the firing rate of OPAB, while enhancing their spectral power density at low frequencies. This work presents original tools, valuable data resources of the composition of adult BDEVs of human origin, and their functional significance in the regulation of neural circuitry.

## 2 Experimental Section

### 2.1 Microfluidics Hardware and Software

**Figure 1:**
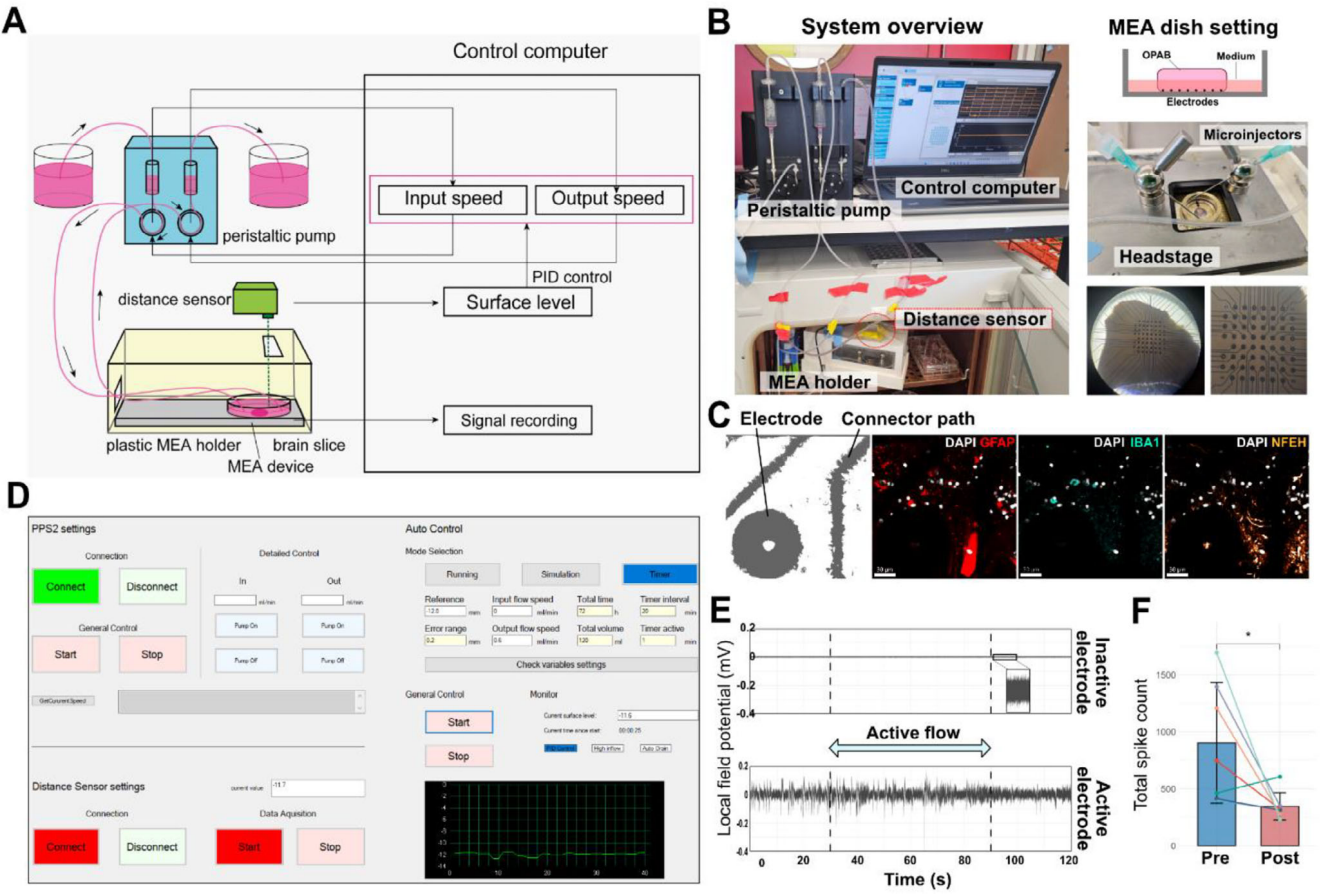
Microfluidic system and control software for automated electrical activity measurement of OPAB and EV collection. A) Schematic representation of the microfluidic‐electrophysiology system. B) Pictures of the platform, showing the dual peristaltic pump coupled to the computer and MEA stage in culture conditions (left panel). The pump has two digital controllers for independent input‐output flow. The MEA stage is contained within a 3D‐printed box (Figure S1 and file S1, Supporting Information) that accommodates the insertion of microfluidic tubing and holds the distance sensor (lower panels). Within the MEA stage, a slice is placed onto 3D electrodes (see Math & Meth) at ALI through precise laser‐based surface level control as illustrated (upper right panel), and shown (middle and lower right panels). C) Snapshots of an OPAB on a MEA chip, fixed, permeabilized, and stained for neurons (NFEH, orange), astrocytes (GFAP, red), and microglia (Iba1, cyan). Nuclei were stained with Dapi (gray). An image with saturated contrasts (left panel) shows the shadow of the electrode that is opaque to light (preventing observation of neurons on the electrode itself, but only the surrounding ones). Images were acquired by confocal microscopy and are representative of at least three fields of view. D) Snapshot of the homemade program enabling users to control the flow either in manual mode (upper left commands) or automatically using a PID‐based controller to adapt input flow and keep a constant predefined level of medium (right commands). The flow level is monitored in real‐time (lower right line graph). E) Examples of a 2 min recording in electrodes showing background activity level (upper panel) and high activity (lower panel). The inset in the upper panel shows a zoom‐in of the background electrical level present in the inactive electrode. The dashed lines represent the interval period during which the flow was on during the recording (active flow, blue arrow). The absence of electrical modulation at start, stop or during flow, highlights the absence of flow‐induced noise. The recordings are representative of more than ten recordings on 60‐electrode MEAs. F) Spontaneous LFP activity was recorded from OPAB that had been pre‐treated with bicuculline and stimulated with glutamate. The LFP signal from the activated OPAB was measured (pre), and then the electrical signal was inhibited for 10 min using a cocktail of TTX, CNQX, and AP5 and LFP was recorded again (post). The graph shows the mean ± SD of the total spike count from seven slices (each line corresponding to a single slice). Statistical comparison was performed with paired Student t‑tests; ^*^ *p* < 0.05.

The hardware consists of a double‐entry peristaltic pump (PPS2, MultiChannel Systems) that creates a flow of medium, a plastic case that houses the dish containing the brain slice and the MEA, and a laser distance sensor (LR‐X50, Keyence) that measures the instantaneous height of media (**Figure** **1**; Figure S1 and obj file, Supporting Information). A distance sensor was used to maintain a precise and constant volume of medium in the dish (difficult to obtain with butterfly pumps alone), which was critical to maintain the OPAB at the air‐liquid interface (ALI). The circulation of the medium and the adjustment of the liquid level were controlled using a programme developed in the laboratory, which was written as a C# Windows Form Application. Given a specific reference medium level, measured using the distance sensor, the program uses Proportional‐Integral‐derivative (PID) control to regulate the outflow and maintain the medium at the reference level. The control of the liquid level was designed in accordance with a standard discrete system PID control (see equation below) where Kp, Ki, and Kd are gain parameters. z represents the z‐transform operator and the error relative to the setpoint at any time index “t” is given by e(t). The gain parameters Kp, Ki, and Kd must be tuned depending on the dish used, the pump characteristics, and the desired error correction rate. In the present setup, it was confirmed that when the gains were set to Kp = 0.3, Ki = 0, and Kd = 0, the liquid level converged to within ± 0.2 mm of the setpoint within 3 s after a disturbance was applied. Given the relatively slow rate of correction required in this case, simple proportional control was sufficient. However, it is noted that the system itself supports full PID control, and can be applied to a wide range of experimental conditions.

(1)

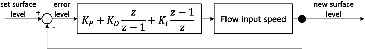




### 2.2 Ethics and Donor Selection

The research presented in the study complies with all relevant ethical regulations. The protocol for the use of *post‐mortem* brain explants for research purposes included in this study was approved by the institutional review board (IRB) of the CHU of Montpellier (approval ID: 202000643) and the French Biomedicine Agency (agreement number PSF20‐025). It permits human brain resection for research purposes during autopsy performed at the forensics institute of the CHU de Montpellier, upon obtaining local prosecutor authorization and family consent.

Excluding criteria were causes of death involving the head and neck (e.g., car crashes, stroke), and previously known severe neuropathology (e.g., epilepsy, neurodegenerative diseases). Altering substances (e.g., alcohol, opiates) were not included in the selection criteria but are reported for each donor (Table S1, Supporting Information). *Post‐mortem* interval for all selected donors was between 0 and 12 h. The data presented in this study originate from donors of aged 23–72 (mean 42) years old.

### 2.3 Brain Explant Preparation and Culture

The brain explants were processed as previously described.^[^ ^17^, ^21^ ^]^ Briefly, upon whole brain was extraction, 2–3 cm thick coronal slice of the primary motor cortex (M1, frontal lobe) was dissected, and immediately transferred for transportation into cold medium containing 1X Neurobasal media (Thermofisher), supplemented with N2 (Gibco), 1X Glutamax (Thermofisher), and 0.5% Penicillin/Streptomycin (Thermofisher), hereafter referred to as N2 media. Under sterile conditions, the *pia mater* was removed, individual folds were isolated and embedded into 3% low‐melting‐point agarose (Thermofisher), and 300 µm thick slices were sectioned using a vibratome (PELCO Easyslicer, Ted Pella). Suitable slices were selected based on the cytoarchitectural integrity of the cortical structure, i.e., presence of white matter and all layers of the gray matter. For culture, brain slices were transferred to an insert lifting a PET membrane with 0.4 µm pores (Sabeu), hanging over 2.5 mL of medium (changed every 2–3 days) in a 6‐well plate, for culturing at the air‐liquid interface (ALI). The OPABs were let to recover from the slicing procedure for at least for 4 days ex vivo. Slices were maintained at 37 °C, 5% CO_2_, and 95% humidity atmosphere. The Organotypic culture of *Post‐mortem* Adult human Brain explants was referred to as OPAB thereafter.

### 2.4 Immunostaining

OPABs were plated on MEAs and fixed after 48 h with 4% paraformaldehyde for 24 h, washed with PBS three times, incubated with blocking solution (0.5% BSA, 0.5% Triton in PBS) O/N, then incubated for 24H with mouse anti Neurofilament H (NF‐H, 1:200, Synaptic Systems #171121), goat anti GFAP (1:400, Novus Biologicals #nb100‐53809), and rabbit anti Iba1 (1:400, Genetex, GTX101495). After three washes with blocking solution and secondary antibody staining for an additional 24 h, samples were cleared for 4 h with Rapiclear 1.52 (Sunjin Labs) and imaged using a Spinning Disk microscope (Olympus) using a long‐distance 20X objective (Leica) to image through MEAs. 3D images were reconstructed with the Imaris software v9.7 and 10.2 (Oxford Instruments).

### 2.5 Extracellular Vesicles Collection

For mass spectrometry, Nanoparticle tracking analysis (NTA), and EV supplementation in electrophysiology experiments, 6 OPABs were used for EV collection from stagnating and microfluidic samples. For stagnating‐based EV collection, 6 OPABs were place in 2 wells of a 6‐well plate and kept at ALI with 500 µL N2 medium in the bottom chamber. Medium was collected after 24 h and wells were washed three times. Cold PBS was added for EV isolation to match the volume of microflow samples and limit centrifugation losses of EVs. For microfluidic‐based EV collection, 6 OPABs were placed on gelified 3% low‐melting‐point agarose dissolved in PBS in a 6 mm dish inside the microfluidics box. Addition of 45 mL of N2 medium was done using a microflow of 600 µL over 1 min every 20 min for 24 h, with the PID‐based controller automatically adjusting the outflow rate to preserve a constant medium level. To accumulate enough material for western blot immunostaining, the collection protocol was adapted by using 18 OPABs on the same surface, and collecting 120 mL of medium over 72 h, with the collected medium kept on ice. Likewise, stagnating‐based EV collection for western blot were obtained from 18 OPABs with medium collected every 24 h for 72 h and kept at 4 °C.

### 2.6 Extracellular Vesicle Isolation

BDEVs were isolated from dissociated OPABs or from Supernatant using either differential Ultracentrifugation (dUC) or size‐exclusion chromatography (SEC), following MISEV recommendations.^[^ ^1^ ^]^ Dissociated BDEVs were prepared from a procedure adapted from.^[^ ^24^ ^]^ Briefly, OPABs were incubated at 37 °C in N2 medium with neutral protease (0.11 DMC U mL^−1^, Nordmark, #S3030111) for 15 min then pipetted up‐and‐down ten times with a cut 1000 µL tip, incubated for 15 more minutes, then mechanically dissociated by pipetting with a 1000 µL pipette until no visible cell clumps remained. After centrifugation at 300 x g for 10 min, the cell pellet was resuspended in RIPA buffer (150 mm sodium chloride, 0.5% sodium deoxycholate, 1% Triton X‐100, 0.1% SDS, 50 mm Tris‐HCl, pH 8.0), constituting the cell lysate, while the non‐pelleted media was used for EV isolation. The dUC protocol consisted of two centrifugations of 2000 x g for 20 min and 10 000 x g for 40′ to pellet the cell debris. The supernatant was recovered and ultracentrifued twice at 100 000 x g to recover the EV pellet, then washed with a second 100 000 x g ultracentrifugation to isolate the EV pellet. For mass spectrometry analysis EVs were resuspended in 50 µL Laemmli Buffer. For Western Blot and NTA analysis, EV pellet was resuspended in 15 µL PBS, and 2 µL were kept for NTA analysis, while 15 µL RIPA buffer was added to the remaining suspension for −20 °C storage followed by immunoblotting. SEC samples were centrifugated at 4000 x g over 100 kDa‐MWCO Amicon Ultra‐15 filters (Millipore UFC9100), then fractionated on qEVoriginal/70nm Gen 2 Columns (Izon, ICO‐70) and carousel, following manufacturer's instructions. EV‐containing fractions 2–4 were pooled and concentrated on Amicon Ultra‐4 columns (Merck Millipore, #C7719). All EV isolation procedures were performed on ice (manipulation) or at 4 °C (centrifugation).

### 2.7 Protein Content and Particle Concentration Measures

Protein content was measured using the micro bicinchoninic acid assay (BCA) kit (Thermofisher) according to manufacturer's instructions. After incubation with the reacting mix, absorption at 562 nm was determined using a spectrophotometer, and the concentration was determined against a standard curve of bovine serum albumin (BSA) dilutions. Concentration of EVs from non‐lysed samples were measured using NTA. Samples were diluted to suitable measuring range to assess the number and hydrodynamic diameter of particles using NTA video tracking of 488 nm laser diffraction on a Zetaview instrument (Particle Metrix).

### 2.8 Western Blot

The concentration of BDEVs obtained from the various conditions was evaluated by NTA to load 5 × 10^8^ BDEVs per well. BDEV preparations were lysed for 30 min on ice in RIPA Buffer, and 2 µL of lysates were added as control for protein detection. Samples were denatured at 95 °C for 10 min after addition of NuPAGE LDS Sample Buffer (Invitrogen), then migrated on Bolt 4–12% Bis–Tris Mini Protein gels (Invitrogen). Proteins were transferred onto nitrocellulose membranes using the iBlot2 Transfer system (Invitrogen) following manufacturer's instructions. Blocking and staining were performed using the Invitrogen iBind Flex Solution Kit on iBind Flex cards (ThermoFisher Scientific). Primary antibodies used were directed against human CD63 (1:500, Thermofisher Scientific, #10628D), CD9 (1:500, Merck Millipore, #CBL162), NCAM‐1 (1:500, BioLegend, #304601), SYNPO (1:500, Synpatic Systems, #163002), or STX1A (1:500, Proteintech, #55164‐1) and chemiluminescence was measured using Clarity or Clarity Max ECL blotting substrate (BioRad) on a Chemidoc MP imaging system (BioRad).

### 2.9 Transmission Electron Microscopy

For TEM analysis 5 µL of sample was dropped on a 100 mesh copper grid formvar‐carbon for 1 min, followed by negative staining on a drop of 1% uranyl acetate in water. The copper grid was dried and analysed using a Tecnai F20 transmission electron microscope at 120 KV at the INM COMET facility.

### 2.10 Mass Spectrometry Analysis

Proteins were loaded on an in‐house poured 4% acrylamide stacking gel. Gel was stained with Coomassie Blue, and the stacking bands were manually excised. Proteins were then reduced using 10 mm dithiothreitol and alkylated using 55 mm iodoacetamide before in‐gel digestion overnight at 37 °C with 0.2 µg of trypsin/LysC (Promega, Madison, USA). Peptides were extracted for 1 h with 90 µL of 60% acetonitrile, 0.1% formic acid. Peptide mixtures were then dried and resuspended in 5 µL water acidified with 0.1% formic acid.

NanoLC‐MS/MS analyses of peptide extracts were performed on a nanoElute 2 LC‐system coupled to a TimsTOF Ultra 2 MS (Bruker Daltonics, Billerica, MA, USA). Mobile phase A was constituted of 99.9% water and 0.1% formic acid, and mobile phase B was constituted of 99.9% acetonitrile and 0.1% formic acid. A sample volume of 1–3.5 µL was loaded onto a Pepsep column (C18‐RP 75 µmx250 mm, 1.5 µm particle size and 100 Å pore size, Bruker) at 120 bars and 2% B. The column was coupled to a 10 µm emitter. This step was followed by reverse‐phase separation at a flow rate of 250 nL min^−1^ and a gradient from 5% to 23% B in 18 min, from 23% to 35% B in 4 min, from 35% B to 90% B in 4 min, and maintained at 90% B for 4 min. The column was further reconditioned using 4 volumes of 2% B respectively. The TimsTOF Ultra 2 instrument was operated in Data Dependent Acquisition‐Parallel Accumulation Serial Fragmentation (DDA‐PASEF) mode with 10 PASEF scans over a mass range from 100 to 1700 m/z leading to a 1.9 s cycle time. The ion mobility range scanned from 0.6 to 1.6 V.s cm^−^^2^ with accumulation and ramp times of 100 ms. Peptides with a minimum intensity of 500 were selected for fragmentation and were automatically added on a dynamic exclusion list for 0.4 min. Collisional induced dissociation (CID) voltage was tuned according to the mobility of the selected ion using a ramp from 20 eV for 0.6 V.s cm^−^^2^ to 59 eV for 1.6 V.s cm^−^^2^.

Raw files were converted to.mgf peaklists using Data Analysis (version 6.1, Bruker Daltonics). Searches were done using Mascot software (version 2.6.2, MatrixScience, London, UK) against a composite database including *Homo Sapiens* protein sequences, which were downloaded from UniProtKB‐SwissProt (17‐01‐2025; 20.321 sequences, Taxonomy ID: 9606) to which common contaminants and decoy sequences were added.

Spectra were searched with a mass tolerance of 10 ppm in MS mode and 10 ppm in MS/MS mode. One trypsin missed cleavage was allowed. Carbamidomethylation of cysteine residues was set as a fixed modification. Oxidation of methionine residues and acetylation of proteins’ n‐termini were set as variable modifications. Identification results were imported into Proline software version 2.3 (http://proline.profiproteomics.fr/)^[^ ^25^ ^]^ for validation. Peptide Spectrum Matches (PSM) with pretty rank equal to one and a minimum length of 7 amino acids were retained. False Discovery Rate (FDR) was then optimized using target‐decoy strategy to 1% at the PSM level using Mascot adjusted E‐value and to 1% at the protein level using mascot modified Mudpit score.

Peptide abundances were extracted thanks to Proline software using a m/z tolerance of 10 ppm. Alignment of the LC‐MS runs was performed using Loess smoothing. No Cross‐assignment was allowed. The best 2+/3+/4+ peptide ion was used to assign an abundance to a peptide. Protein abundance was then computed by summing the abundance of all specific peptides. A complete proteomics dataset was deposited to the Pride repository^[^ ^26^ ^]^ and is accessible under PXD064092.

### 2.11 Local Field Potential Recording

OPAB were plated on 3D MEA (Multichannel System), containing an array of 60 TiN‐coated conical electrodes of 12 µm diameter active site and 80 µm height for sensitive LFP recording LFP measurement, and stimulation of the tissue (Multi Channel Experimenter software) as previously described.^[^ ^21^ ^]^ Briefly, the OPAB were maintained in 150 µL BrainPhys aCSF (StemCell Technologies), supplemented with N2 and Glutamax. MEA chips were mounted onto a MEA2100‐Mini headstage (Multichannel Systems) connected to a digital amplifier (Multichannel Systems). Headstage was shielded using tin foil and placed in an incubator at 37 °C, 5% CO_2_, and 95% humidity atmosphere for recording. LFP data was acquired at a 10 kHz sampling rate.

For electrophysiology experiments, BDEVs were first collected from 6 OPABs with the microflow method artificial cerebrospinal fluid (aCSF, Brainphys (Stemcell # 05790) supplemented with N2, Glutamax and 1%P/S). Then OPABs on MEAs were mounted in the microfluidic system, with a periodic flow of non‐supplemented aCSF (control condition), aCSF supplemented with half of the donor‐matched BDEV preparation (EV Condition), 20 µm of GW‐4968 (MedChem Express, #HY‐19363) (GW Condition), or GW‐4968 and half of the donor‐matched BDEV preparation (GW‐EV condition). LFP of all 60 electrodes was recorded for 2 min every 20 min for 12 h between microflow occurrences.

### 2.12 Electrical Activity Analyses

Data was analysed using the Firelearn Software (v9.0.0‐alpha, manuscript in preparation). For power density‐based analyses, the data was split into 10 s fragments, and Fast‐Fourier Transformation (FFT) was performed to obtain power densities for frequencies between 0.2 to 300 Hz (by 0.1 Hz). All electrodes were considered for spectrogram representations and wave domain analysis, and average frequency power densities were represented over time. For the random forest classification (RFC), the 20 most active electrodes (presenting the highest standard deviation) were selected, and then the dataset was split into train (70%) and test (30%) datasets The RFC was trained to discriminate between the conditions, and validated against overfitting with a five‐fold cross‐validation. For activation of spike count, LFP was recorded in the presence of 40 µm bicuculine (MedChemExpress, # HY‐N0219) for 3 h and subsequently stimulated with 100 µm glutamate (MedChemExpress, #HY‐14608) for 10 min (activation). The spike inhibition was performed by adding a cocktail of 50 µm tetrodotoxin (TTX; Acros Organics, #13187663)), 50 µm cyanquixaline (CNQX; Sigma–Aldrich, #C127‐5MG), and 50 µm (2R)‐amino‐5‐phosphonovaleric acid (AP5; MedChemExpress, # HY‐100714A) for 10 min prior to recording. Spikes were detected using a 10‐ms rolling window detecting values above 5.5 standard deviation (SD).

### 2.13 Statistical Analysis

Data is presented as mean ± SEM, unless indicated otherwise. No outliers were removed. Proteomic data was handled using Perseus 2.1.1 (MaxQuant) and R. Quality filtering retained only 1919 proteins expressed in > 75% of samples in at least one condition. Imputation was performed using the ImputeLCMD package setting undetected values at the 2.5% lower quantile. To compare dissociated conditions, the Trimmed Mean of M‐values (TMM) method implemented in the EdgeR package^[^ ^27^ ^]^ was used to generate adjusted p‐values and differences. For supernatant samples, as input protein levels were not normalized, proteins were ranked for each sample based on relative expression levels, and protein ranks were compared between conditions. Ontology analyses were performed using GSEA software.^[^ ^28^ ^]^ Synapse‐specific ontology was evaluated using SynGO.^[^ ^29^ ^]^ Venn diagrams were generated using InteractiVenn.^[^ ^30^ ^]^ For statistical comparisons of spike counts and frequency domain power densities, multiple *t*‐tests with Bonferroni correction for False Discovery rate (FDR) were used, with post‐hoc Tukey‐HSD multiple comparisons. For size distribution, non‐linear Gaussian best fits were calculated and used to perform One‐way ANOVA with Holm‐Sidak post‐hoc multiple comparisons. For Zeta potential comparisons, a Kruskal–Wallis test was performed with Dunn's post‐hoc multiple comparisons. For protein abundance, One‐way ANOVA with Holm‐Sidak post‐hoc multiple comparisons were used. For RFC statistical significance, the prediction for each tested condition was compared to the random assignment hypothesis, corresponding to 33.3% per class (as three options were trained), using a chi‐square test.

## 3 Results

### 3.1 Microfluidic System Development for Injection/Collection on OPABs Seeded on a 3D MEA

The OPAB culture requires to be maintained at the ALI, which is routinely achieved by using a transwell culture system.^[^ ^17^ ^]^ However, MEA recording is not available on transwell membranes, and to combine ALI and electrophysiology, we add a shallow layer of media to cover the bottom of a MEA chip without submerging the brain slice. This culture method is prone to rapid evaporation at 37 °C, even in high humidity atmosphere. To overcome this challenge, we designed and built a microfluidic system composed of two peristaltic pumps injecting media on one side of the dish, and sucking media on the other side allowing a slow flow rate (600 µL min^−1^; Figure 1A). In order to compensate for the loss of media over time through evaporation, we equipped this system with a laser distance sensor (with 100 µm precision) that allows the automatic adjustment of input media in order to maintain a constant surface level. The whole system is integrated within a 3D printed bow that fits the MEA2100‐Mini‐system stage (Multichannel Systems), holds the input/output syringes and the distance sensor, includes a transparent window, and holes to let tubulatures go in and out (Figure 1B; Figure S1, and 3D printing file available in, Supporting Information). The OPAB were covering the whole electrode array and displayed typical neural cells, in direct contact with the electrodes (Figure 1B,C).^[^ ^21^ ^]^ This integrated system is operated via a graphical user interface (GUI) controlling a home‐made algorithm integrating a proportional integral derivative (PID), smoothly adjusting flow (Figure 1D). The presence/absence of flow showed no difference in the electrical activity of electrodes recording active signal, while background levels also remained unchanged (Figure 1E), and treatment with antagonists of neurotransmitter receptors confirmed that the detected signal originates from neuronal activity (Figure 1F).

### 3.2 Characterization of BDEVs Secreted by OPAB

**Figure 2:**
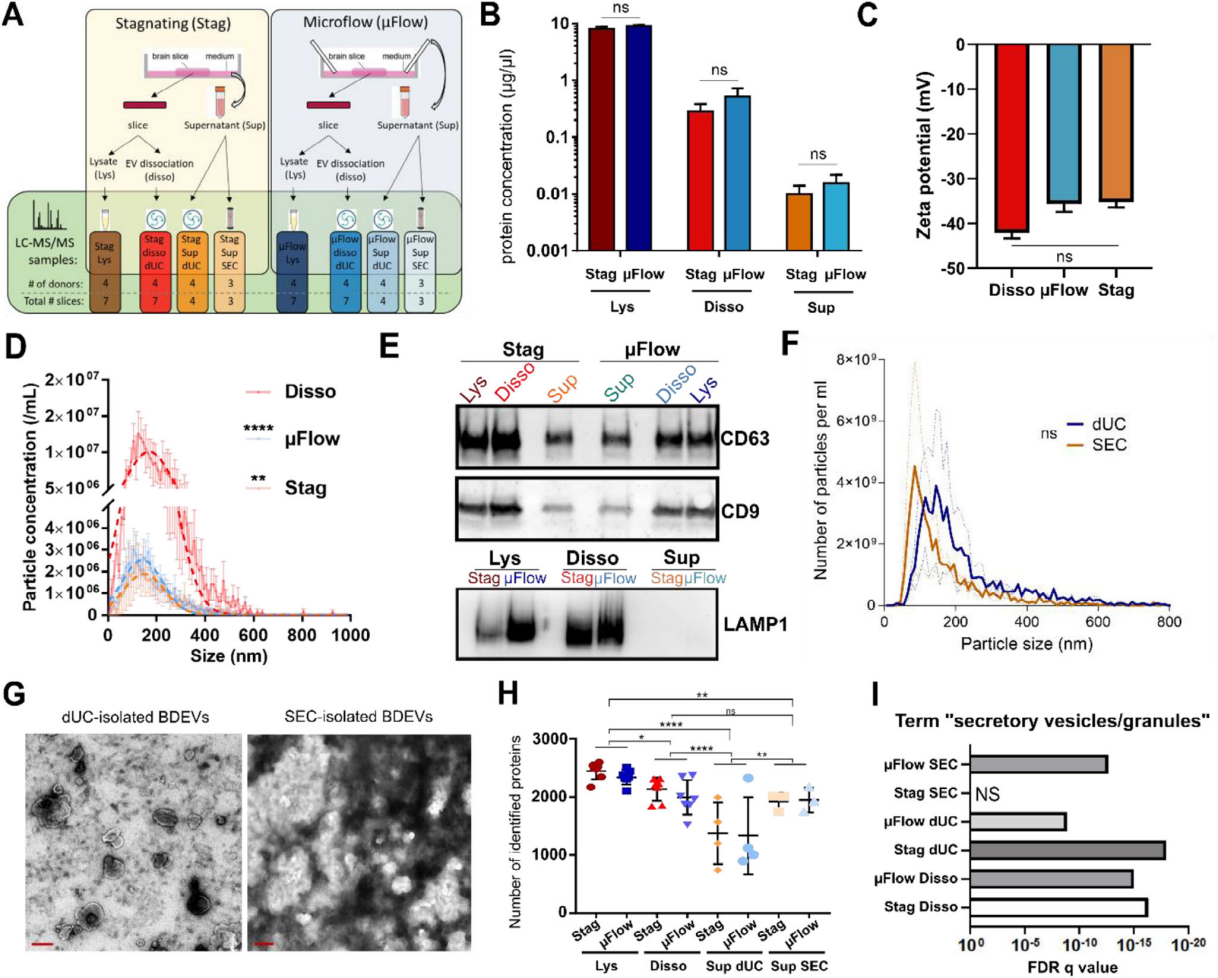
Characterization of BDEVs from OPAB. A) Diagram of BDEV isolation conditions, comprising two settings: microflow (µFlow) or stagnating (Stag) conditions; three sample origins: tissue lysate (Lys), tissue dissociation (Diss), and supernatant (Sup); and two purification methods: differential ultracentrifugation (dUC) and size‐exclusion chromatography (SEC). The number of donors and slices analysed per condition is shown in the bottom section of the scheme. B) Protein concentration of the different samples measured by BCA showing the mean ± SEM (*n* = 4 experiments from four donors; ns = non‐significant statistical difference between Stag and µFlow samples for each condition). C) Zeta potential using the ZetaView instrument was measured for the indicated conditions, and no statistical difference was observed (*n* = 3 experiments; Kruskal‐Wallis with Dunn's post‐hoc multiple comparisons; ns = non‐significant statistical difference). D) NTA of dUC‐isolated samples showing concentration as a function of BDEV size. The disso sample represents average of Stag and µFlow BDEV collection. Data are mean ± SEM (*n* = 3 experiments from three donors; One‐way ANOVA of best‐fit for nonlinear Gaussian fit; ^****^: *p* < 0.001, ^**^: *p* < 0.01). E) Western blot stainings of EV markers CD63 and CD9, and contaminant Lamp1 in dUC‐isolated samples. Images are representative of two experiments. F) NTA of dUC‐isolated versus SEC‐isolated BDEVs collected from OPAB showing particle concentration as a function of size. Data are mean ± SEM (*n* = 2 experiments from two donors; ns = non‐significant statistical difference). G) Snapshots of BDEVs from the supernatant of OPAB under µFlow, isolated by dUC (left panel), or SEC (right panel). The images were acquired by transmission electron microscopy upon negative staining, and are representative of BDEVs isolated from two individual donors. Scale bare = 200 nm. H) Number of proteins identified by mass spectrometry for each individual slice. Data are mean ± SEM of 3 to 7 slices as indicated in Figure 2A. Statistical analysis was performed using one‐way ANOVA with Holm‐Sidak post‐hoc multiple comparisons *p*‐value < 0.05 (^*^), 0.005 (^**^), and 0.001 (^****^). I) FDR q value of the enrichment of ontology terms “secretory vesicles” or “secretory granules” based on the hundred most abundant proteins in each condition. NS = non‐significant.

We used our automated microflow system to harvest BDEVs secreted by OPAB at frequent intervals without the experimenter's intervention. Here we setup the automated collection of 600 µL of OPAB supernatant (Sup) every 20 min for 24 h and compared the composition of microflow‐collected BDEVs (referred to as MF) to the stagnating supernatant of OPAB that was not exposed to microflow (referred to as Stag; see scheme in **Figure** **2**A). To isolate the BDEVs from MF or Stag supernatant, we employed two classic EV isolation methods: differential ultracentrifugation (dUC) or size‐exclusion chromatography (SEC). Alternatively, instead of collecting BDEVs from Sup, we also isolated BDEVs from within brain slices using a recent protocol that combines mechanical and enzymatic dissociation of OPAB (referred to as Disso), followed by differential ultracentrifugation (dUC).^[^ ^31^ ^]^ These methods aim to study more particularly small EVs (< 200 µm) without differentiating between their endosomal (exosome) or plasma membrane (ectosome) origin.

The amount of proteins recovered after dUC was over a log more in the Disso than in the Sup conditions, regardless of the application of flow (Figure 2B), indicating that the Disso strategy retrieves more biological material. Nanoparticle tracking analysis (NTA) confirmed the presence of BDEVs in all conditions, with non‐significant differences between the zeta potential of the particles (Figure 2C). Particle size was ranging between 40 and 250 µm for Sup conditions (µFlow and Stag), consistent with previous work,^[^ ^32^ ^]^ while larger vesicles up to 500 µm were also recovered in the Disso condition (Figure 2D). All conditions revealed the presence of typical EV‐associated proteins, including CD63 and CD9 (Figure 2E). In contrast, the lysosomal marker Lamp1 was found in the Disso conditions but was absent in the BDEVs prepared from supernatants (Figure 2E, lower panel), indicating that the Disso isolation procedure is not as clean as the supernatant approach, and thus, may carry over some non‐EV contaminants. Examination of the dUC and SEC isolation methods by NTA showed a non‐significant difference in particle size range, although dUC tends to retrieve slightly larger vesicles (Figure 2F). The NTA data of BDEVs isolated by dUC were consistent with the size of the vesicles observed by transmission electron microscopy (TEM; Figure 2G). SEC‐isolated BDEVs also had overall consistent sizes, but TEM revealed a significant association of the vesicles with a darker staining suggestive of free protein carry over (Figure 2G).

### 3.3 Proteomic Analyses of BDEV Composition According to Sample Preparation

To unbiasedly analyse the composition of BDEVs collected from the various conditions, we next performed liquid chromatography with tandem mass spectrometry (LC‐MS/MS) using a highly sensitive TimsTOF Ultra 2 instrument on 42 samples originating from four OPAB donors (Figure 2A; Table S1, Supporting Information). With this approach, we were able to detect between 742 and 2596 unique proteins depending on the samples (Figure 2H and protein abundance for each condition in Table S2, Supporting Information). Of note, the amount of proteins loaded on the LC‐MS/MS system could not be normalized between samples as the Sup conditions (µFlow and Stag) contained much less proteins than the BDEVs isolated from dissociated tissues (Disso). For comparisons, we have ranked protein expression in each sample, and used rank average across samples from the same condition as a proxy for relative importance of a given protein.

Ontology terms associated to the hundred most abundant proteins per condition were analysed (Table S3, Supporting Information). The terms “secretory vesicle” and “secretory granules” were associated with high significance to all conditions, except for BDEV isolation by SEC from Stag Sup (Figure 2I). We found that OPAB exposed to a microflow (µFlow) or not (Stag) produced BDEVs with protein similarity of 81% (95/117) for Disso, 59% (77/129) for SEC, and 18% (30/165) for dUC (Figure S2, Supporting Information). These data indicate that the methods of OPAB culture and BDEV isolation significantly affect their composition. Indeed, when comparing the three isolation methods (Disso, dUC, or SEC), either in the µFlow or Stag conditions, most shared proteins were associated to catabolic processes (Figure S3 and Table S4, Supporting Information), which may originate from non‐specific cell debris.

### 3.4 BDEVs Harbour Synaptic Components

**Figure 3:**
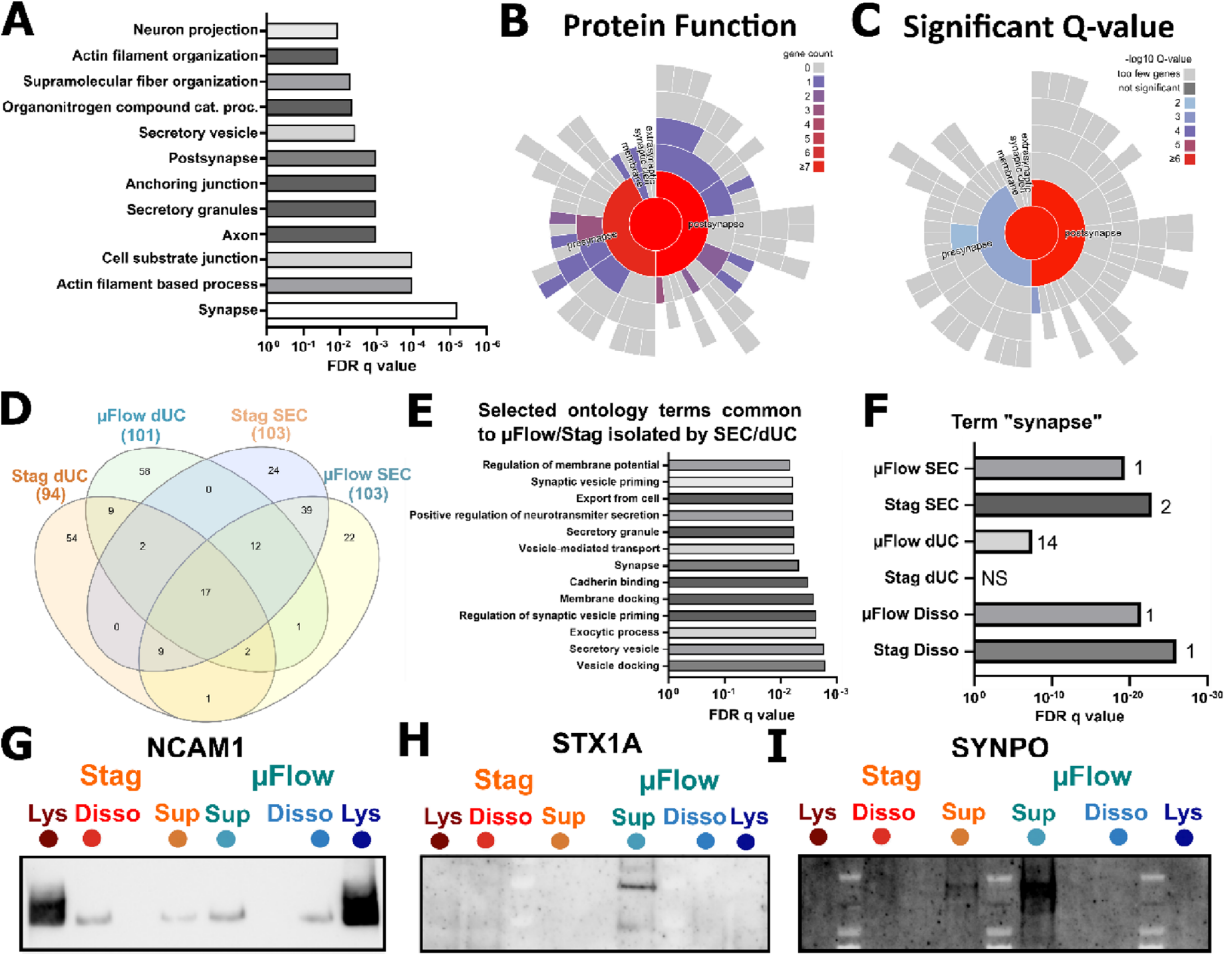
BDEVs are enriched in synaptic components. A) Bar chart showing the FDR q value of the most significant ontology terms associated with the proteins enriched in the Disso‐µFlow condition compared to the Disso‐Stag condition. B,C) Analysis of synapse‐associated protein localisation, function and significance enriched in the Disso‐µFlow condition compared to the Disso‐Stag condition using SynGO. D) Venn diagram showing the overlap of most abundant proteins identified in BDEVs isolated from supernatants. E) Selected ontology terms associated with the BDEV proteins shared by the four conditions. F) FDR q value of the enrichment of ontology terms “synapse” based on the hundred most abundant proteins in each condition. The number at the end of each bar represents the ranking of the term “synapse” for each condition. NS = non‐significant. G–I) Western blot stainings of synaptic proteins NCAM1, SYNPO, and STX1A in the indicated conditions. Each lane was loaded with 5 × 10^8^ BDEVs.

The conditions originating from Disso yielded more proteins than Sup conditions, and thus, they were analysed separately in the following analyses to avoid biases. BDEV from the Disso‐µFlow condition had enriched composition of proteins associated to “secretory granules” and “secretory vesicles” compared to the Disso‐Stag condition (**Figure** **3**A), suggesting that application of microflow favors BDEV recovery. Interestingly, BDEVs from the Disso‐µFlow condition exhibit higher levels of synaptic protein composition compared to the Disso‐Stag condition (unpaired Student's *t*‐test p value = 6 10^−10^ (Figure 3A–C). As it was previously shown that BDEVs harbour synaptic components,^[^ ^6^, ^23^ ^]^ our data highlights the qualitative superiority of BDEV collection under flow conditions.

The same analysis on BDEVs collected in the Sup did not show significant differences between µFlow and Stag conditions, likely limited by the low abundance of identified proteins (Figure 2B). Indeed, the different isolation methods presented heterogeneous profiles, with only 17 proteins common to all four conditions (Figure 3D). These proteins were associated with pathways, including secretory granule and synaptic processes, although with low FDR q value (Figure 3E; Table S4, Supporting Information). However, when analysing the most expressed proteins in each condition individually, “synapse” was one of the most significant terms that emerged from all conditions, except for BDEV isolation by dUC from the Stag Sup (Figure 3F). This further suggests that evaluating BDEV composition by collecting stagnating supernatant of brain explants may result in the loss of some proteins or EV subsets with short lifespans and/or quickly re‐uptaken.

To confirm the enrichment of synaptic components in BDEVs, western blot analysis was performed for synaptic proteins Syntaxin‐1A (STX1A), Neural cell adhesion molecule 1 (NCAM1), and Synaptopodin (SYNPO), which play major roles in presynaptic vesicular docking, synaptic plasticity, and cognition.^[^ ^33^, ^34^, ^35^, ^36^ ^]^ NCAM1 was detected in all conditions, further confirming that regardless of the method, synaptic proteins can be packaged onto BDEVs (Figure 3G). We could also confirm the presence of SYNPO and STX1A in some of the samples (Figure 3H,I), including into the Sup dUC condition, consistent with the ontology analysis for this condition (Figure S4, Supporting Information). Of note, due to the inter‐donor and inter‐slice heterogeneity, and given the different yields retrieved in each condition, this data could not be robustly quantified to compare between the conditions, and should be taken as the qualitative confirmation that synaptic proteins are found in BDEVs.

### 3.5 BDEVs Modulate Electrical Activity of Human Brain Explants

**Figure 4:**
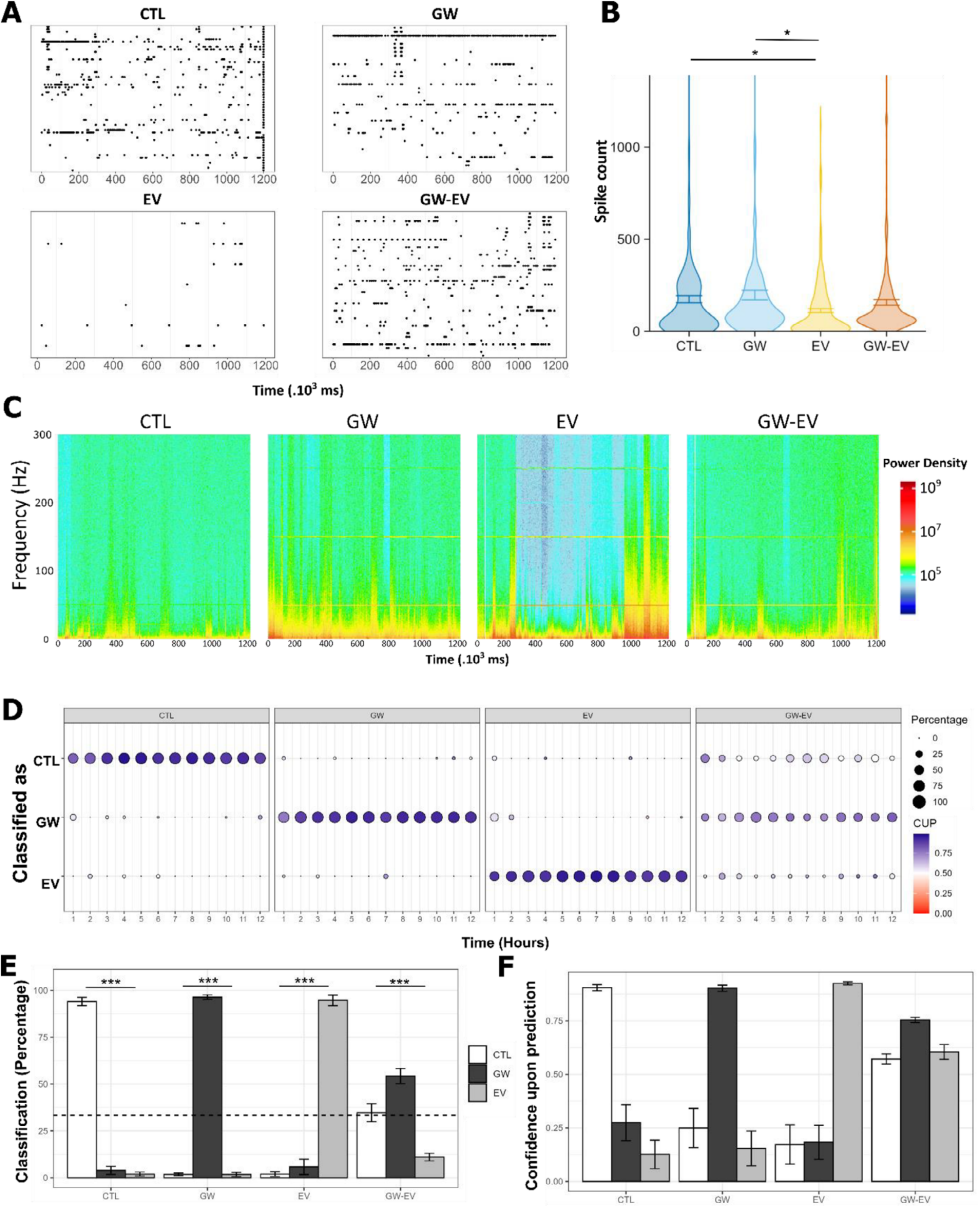
BDEVs modulate the neuronal activity of human brain explants. A) Raster plot of the detected spontaneous spike activity in a representative 2 min recording of OPAB with microfluidics‐mediated record change. B) The graph represents the mean ± SD of detected spike counts per electrode with n = 5 experiments from 5 donors and multiple t‐test with Bonferroni correction, and post‐hoc Tukey‐HSD multiple comparisons *p* value < 0.05 (^*^). C) Spectrogram of the intensity of the signal at frequencies 0–300 Hz as a function of time (12 h total) for each condition. The spectrograms are representative of at least three slices. D) Classification of LFP signals of control (CTL), GW, EV and GW‐EV conditions using a machine learning framework (see Experimental Section) trained to recognize CTL, GW‐4968 and EV conditions from four donors. E,F) Predicted class repartition E) and confidence upon prediction F) for each condition. The bars represent the mean ± SEM from four donors. The *p* value < 0.001 (^***^) calculated in E was obtained from a chi‐square test against random assignment hypothesis represented with a dotted line.

Because of the link between BDEVs and synapses, we next investigated the functional impact that BDEVs may exert on the local field potential (LFP) of OPAB. To this end, we collected and isolated BDEVs using the Sup‐dUC µFlow procedure (as the preparation was cleaner than SEC isolation as observed in Figure 2G), and evaluated their impact on the electrical activity of BDEVs over time using a 3D microelectrode array as previously described.^[^ ^21^ ^]^ Surprisingly, we observed that both BDEV‐supplemented media decreased the overall electrical activity of the OPAB (**Figure** **4**A,B). In contrast, chemical inhibition of EV secretion using GW4869 (GW), a sphingomyelinase inhibitor commonly used to inhibit exosome secretion, did not show a significant effect on OPAB LFP. The simultaneous exposure of OPAB to GW and isolated EVs (hereafter referred to as GW + EV) rescued the dampened spontaneous firing rate observed in EVs alone. Decomposition of electrical signal represented as spectrograms (frequency as a function of time) evidenced that the power density was not uniform. For instance, BDEV‐treated samples showed high signals at time points 1000 to 1200 as represented by the increased red/yellow color‐coding (Figure 4C), while a very weak power density was observed at frequencies > 50 hz and time points 300 to 1000, as represented by the cyan/blue color‐coding. This observation indicates that EVs cannot be considered as a unique entity exhibiting a single phenotype, but rather the convolved modulation of electrical activity that are depending on specific location, time, and frequencies.

BDEVs are produced by different cells, in different proportions, and have different compositions. Analysis of cell type origin of BDEVs, based on the presence of specific markers previously identified using stem cell‐derived neural cells,^[^ ^16^ ^]^ showed that BDEVs secreted by OPAB exhibit relative high amounts of a subset of markers from neurons, astrocytes, microglia, and oligodendrocytes (Figure S5, Supporting Information). This observation is not quantitative as different proteins will be identified with different sensitivity by LC‐MS/MS, but it provides indirect confirmation that OPAB‐derived BDEVs originate from various neural cells, conferring them with interwoven roles.

The pleiotropic properties of BDEVs pushed us to further decompose their impact on the brainwaves of specific frequencies usually observed by EEGs. We found that the profiles of the various conditions were heterogeneous (Figure S6A–G, Supporting Information), likely due to the inter‐slice and inter‐donor variability. The highly sensitive regulation of electrical activity is well‐illustrated by the significant changes induced at all frequencies upon stimulus addition. Interestingly, however, while relatively negligible differences were observed between GW, EV, and GW+EV conditions (Figure S6A–F, Supporting Information), high frequency oscillations (HFO; ranging between 80 and 600 Hz) were decreased upon EV addition compared to GW and GW+EV (Figure S6G, Supporting Information), suggesting that BDEVs may negatively regulate ripples typically observed in epileptiform activity.^[^ ^37^ ^]^ This observation again points out toward a dual role of BDEVs, able to increase or decrease the electrical activity of OPAB depending on the frequency oscillations analyzed.

To analyse the impact of EVs on OPAB electrical activity, we developed a pipeline to uncouple their global effects using a custom‐made machine learning algorithm (see^[^ ^17^ ^]^ for details) able to unbiasedly classify the specific changes associated with each condition (Figure 4D–F). Training the algorithm to recognize Mock, GW‐, and EV‐treated LFPs over time allowed accurate segregation of each condition based on their LFP signature, with overall percentage classification and confidence upon prediction (CUP) over 90% and 0.83 respectively. Classification of the LFP originating from the GW+EV condition mainly classified either as Mock (32%) or as GW (57%), and significantly less often as EV (11%). As GW is proposed to mostly inhibit the release of EVs of intracellular origin (exosomes), and less the ones originating from the plasma membrane (ectosomes), our data suggests that various subtypes of BDEVs are responsible for the differential regulation of the electrical signal in OPAB. Further investigations are needed to uncouple the specific effects of exosomes, ectosomes, and all their subpopulations on electrophysiological features of human neural systems.

## 4 Discussion

The emergence of brain‐derived extracellular vesicles (BDEVs) as critical mediators of neural communication has reshaped our understanding of brain plasticity and synaptic homeostasis.^[^ ^5^ ^]^ However, a recurring issue in EV biology is the wide heterogeneity of their composition, which is multifactorial, including sample origin, model and species system, EV collection and isolation procedures, and the analytical method employed. Here, we provide a comparative analysis of BDEV composition obtained from adult human cortex from four donors, using two collection procedures and three isolation methods, and analyzed them by ultra‐sensitive mass spectrometry. We highlight the importance of the methodology employed in the outcome and associated interpretations, and selected the most relevant approach to further decipher the role of BDEV in regulating synaptic transmission.

Microfluidic MEA systems have been developed to study the electrophysiology of 2D cultures, and have been more recently applied to 3D neuronal networks.^[^ ^38^ ^]^ However, ALI culture is being recognized to provide ideal conditions for brain organoids and brain explant cultures,^[^ ^17^, ^39^, ^40^ ^]^ which is incompatible with current microfluidic devices. Our OPAB model cannot be submerged by media, which is why we developed an open‐chamber microfluidic platform with precisely controlled surface level in real‐time.

Microfluidic platforms are being increasingly developed and used for EV purification and analysis.^[^ ^41^, ^42^ ^]^ In contrast, EV collection is traditionally performed in a passive manner, letting cells secrete EVs in the extracellular media for several days. Such accumulation‐based EV collection suffers, however from critical limitations. Indeed, these approaches disproportionately capture long‐lived EVs with stable surface markers, while the rapidly internalized subpopulations of EVs may be underrepresented despite high abundance. Our microfluidic platform circumvents this by enabling frequent EV collection (every 20 min), while performing electrophysiological recording.

The isolation of BDEVs from dissociated tissues provided us with very high yields compared to supernatant‐recovered methods. This difference could be explained by a greater number of “stuck” BDEVs contained within the slices compared to the ones released in the supernatant of OPAB. However, the composition of these particles suggests that the preparation does not contain solely BDEVs, but other cellular components, as indicated by the presence of Lamp1 and the enrichment of catabolism‐associated ontology. Hence, while the harvest of “stuck” EVs remains very relevant, methodologies for BDEV collection based on tissue dissociation should be further optimized in the future to better represent the diversity of BDEVs while avoiding contaminant co‐isolation. We also observed significant differences of BDEV composition when isolated from SEC or dUC. This may be due to some carryover proteins observed by TEM in the SEC‐based preparation of BDEVs compared to dUC‐isolated BDEVs.

The three untargeted BDEV isolation methods we employed yielded a broad range of BDEV concentrations, hindering measured abundance‐based differential analysis of the identified proteins by mass spectrometry. Therefore, we analyzed the data based on protein ranking within a given condition. With this approach, we found that the terms linked to “synapse” was among the most prevalent in most conditions tested, highlighting that synaptic proteins are significantly enriched in BDEVs, regardless of the isolation procedure. Of note, while we focused our study on three untargeted BDEV isolation approaches, we did not explore the use of commercial kits for EV isolation based on immune‐retention of markers such as CD63, CD9, and CD81. Such methodology has the advantage of targeting some specific BDEV populations, but may in the meantime miss some BDEVs that would be negative for these traditional markers.

The heterogeneity of EVs is well‐recognized, and depends on the methodological isolation procedure, as well as the biological origin (tissue type and species), as previously exemplified.^[^ ^43^ ^]^ Hence, while using monoculture of cells grown in 2D provided significant insights onto the specificities of the produced EVs based on their cellular origin, it does not represent the complex interplay occurring between cells that necessarily regulates the diversity of EVs. This is even more relevant when discussing about EV function, as different cell types may produce EVs with opposite actions, as previously shown in the context of Herpes Simplex virus infection.^[^ ^44^ ^]^ Thus, similarly to the role of the various cytokines during inflammation, we hypothesize that physiological tissue functions are likely to be regulated through the fine‐tuned balance of a complex mixture of EV subtypes. Investigating the BDEV population as a whole is critical to decipher their role at the macroscale, in order to then orientate research on the functional correlations between EV subtypes and cell type origin.

Comparison of supernatant µFlow‐ and Stag‐based collection highlighted that BDEV composition was modified. We observed an enrichment of synaptic proteins in BDEVs that is consistent with recent literature demonstrating the presence of synaptic components on BDEVs.^[^ ^6^, ^23^ ^]^ We hence hypothesized that BDEVs have a functional impact on the electrophysiological activity of OPAB. We found that BDEVs significantly dampen the spontaneous firing rate of OPAB. While surprising at first, this is consistent with previous observations showing that inhibitory postsynaptic current (IPSC) is increased upon exposure of mouse hippocampal neurons to EVs.^[^ ^23^ ^]^ By coupling N‐SMase inhibition (blocking exosome biogenesis) via GW4968 with functional partial rescue via isolated BDEVs, we uncover a link between EV‐laden synaptic proteins and network activity maintenance. It should be noted that GW treatment is not specific to the inhibition of exosome release, and it has been shown that it can also interfere with endocytic processes.^[^ ^45^ ^]^ Hence, further experiments are needed to fully address the role of exosomes and their contribution to the synaptic function of BDEVs.

Thanks to the development of our ALI‐microfluidic platform combined to the electrophysiological recording of cortical explants, we provide a valuable resource regarding the composition of brain EV of human origin. We were also able to highlight for the first time to our knowledge the modulatory role of BDEVs in physiologically relevant settings. The open‐access list of enriched proteins in each collection and isolation method shall drive future mechanistic studies on the role of BDEV in synaptic plasticity in health and disease.

## Conflict of Interest

The authors declare no conflict of interest.

## Supporting information



Supporting Information

Supplementary Table 1

Supplementary Table 2

Supplementary Table 3

Supplementary Table 4

Supplementary Table 5

Supplementary Table 6

Supporting Information

## Data Availability

The data that support the findings of this study are available in the supplementary material of this article.
