# Supplementary material for: Assessing the Nature of Human Brain‐Derived Extracellular Vesicles on Synaptic Activity Via the Development of an Air‐liquid Microfluidic Platform
Source: Adv Sci (Weinh). 2025 Oct 5;13(2):e11194. doi: 10.1002/advs.202511194 (PMC12786325; doi:10.1002/advs.202511194)
Supplement: Supplementary file 1 — Supporting Information [file ADVS-13-e11194-s001.docx]

**Supplemental materials**

**
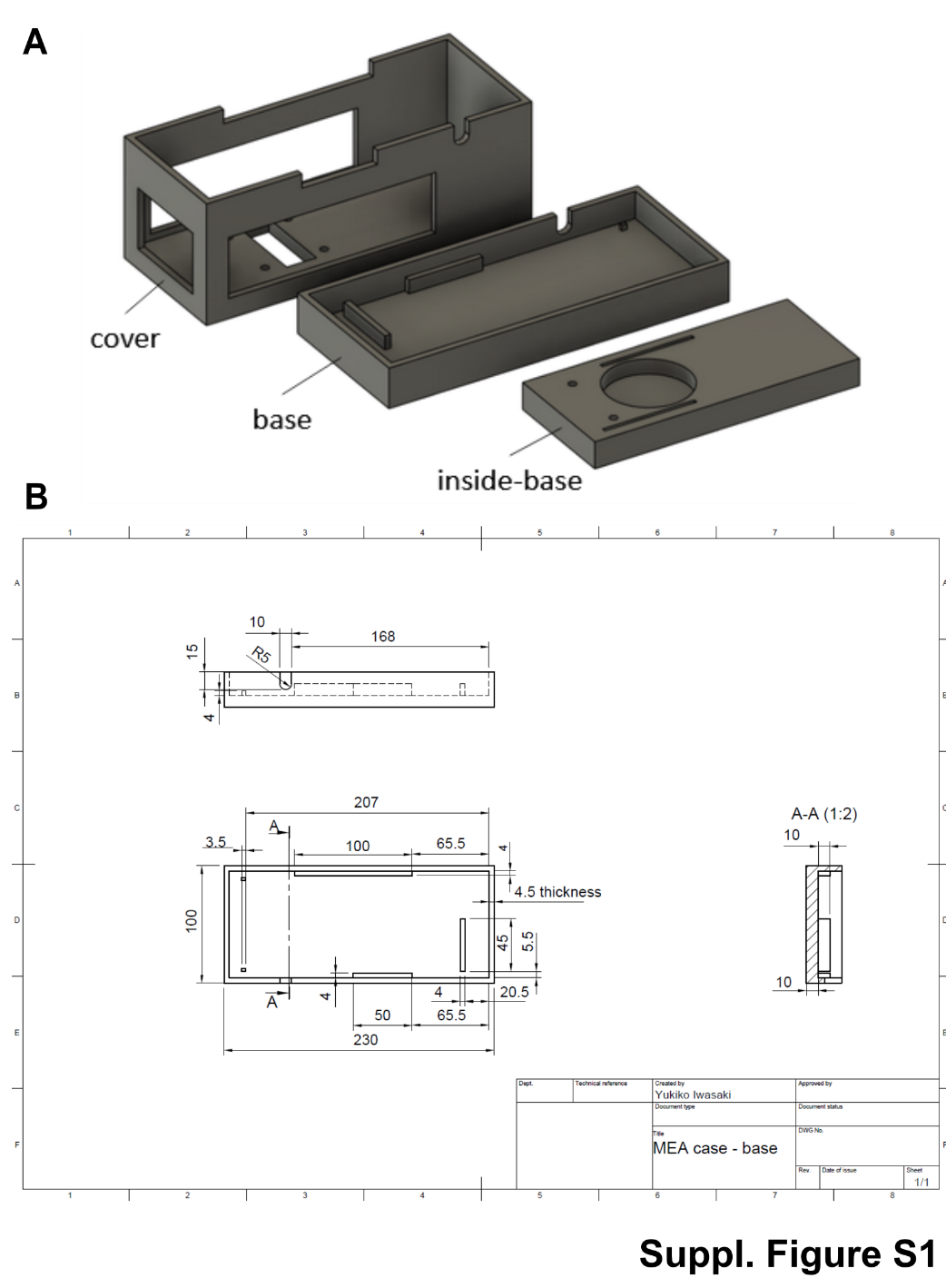
**

**Suppl Figure S1.** Three-dimensional view (**A**) and dimensional drawing (**B**) of the 3D-printed box that integrates the MEA stage, microfluidics, and sensor. The file to 3D-print this box is available in Suppl. File S1.


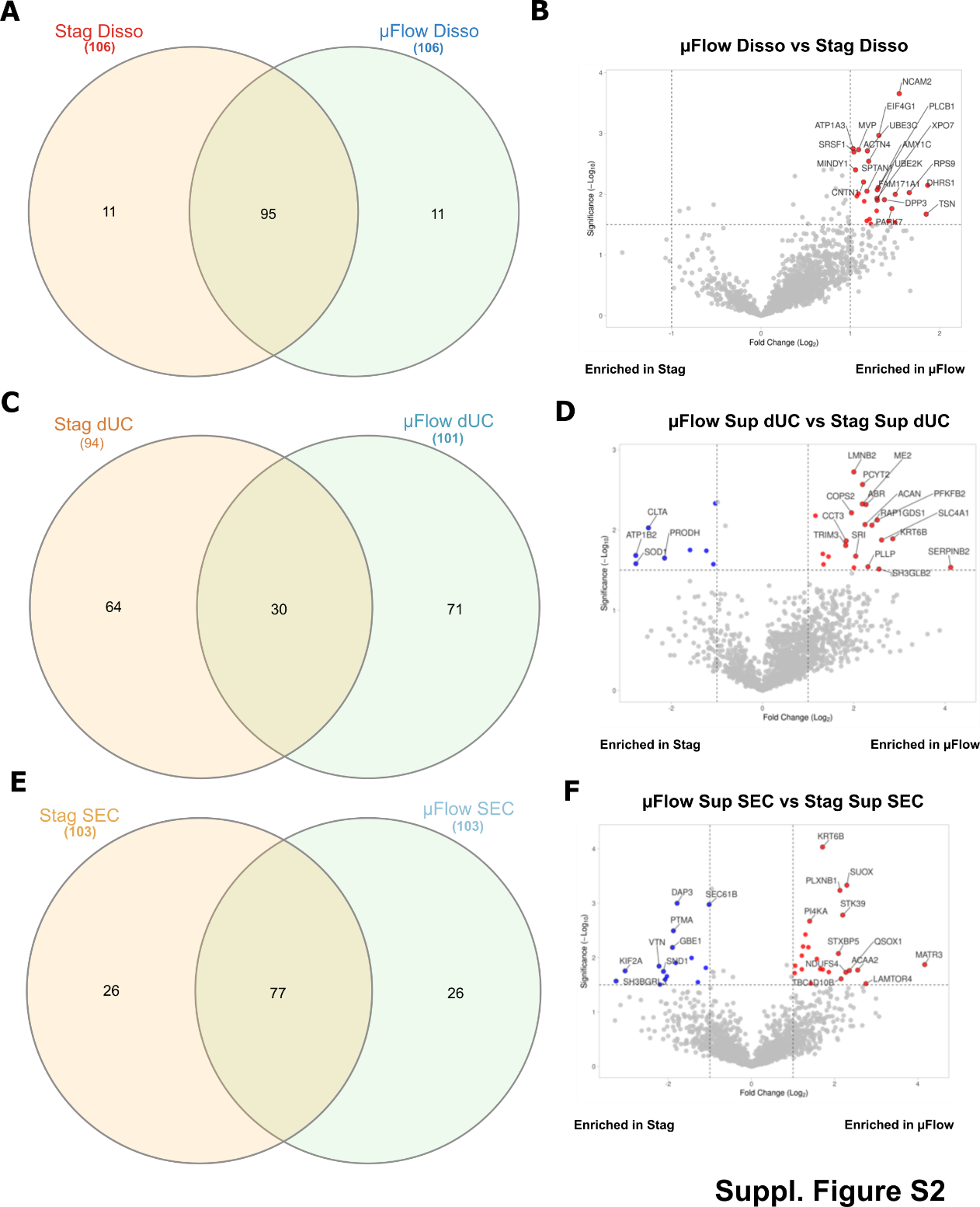


**Suppl Figure S2. Comparison of BDEV protein expression between Stagnating and microfluidics conditions.** Venn diagram (**A**, **C**, **E**) of the top 100 ranked proteins and Volcano plot of differentially expressed proteins (**B**, **D**, **F**) in Disso, dUC, and SEC, respectively.

**
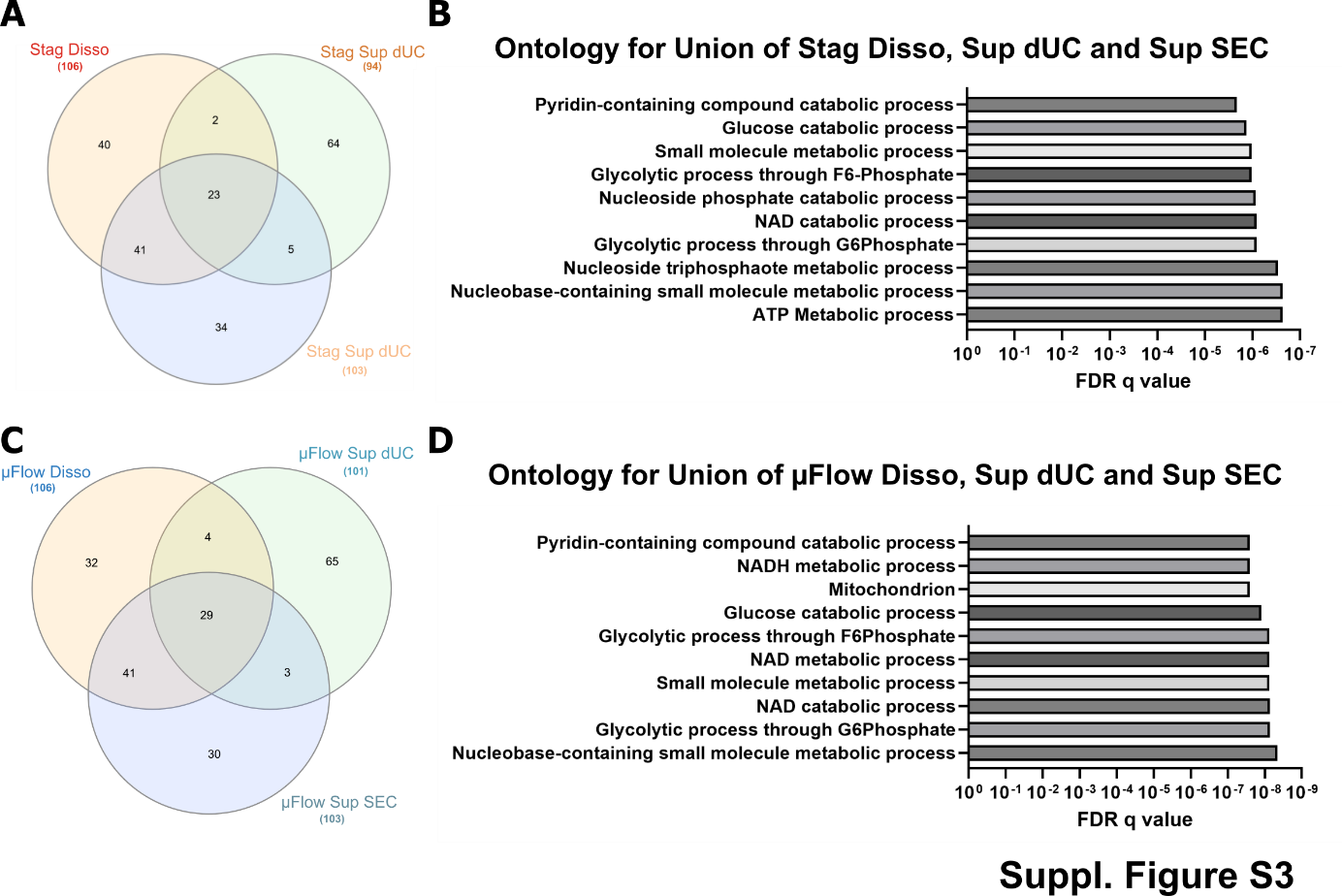
**

**Suppl Figure S3. Comparison of BDEV protein expression between isolation methods.** Venn diagram (**A**,**C**) of the top 100 ranked proteins and Gene ontology of common proteins (**B**,**D**) between the isolation methods for the Stag and µFlow conditions, respectively.


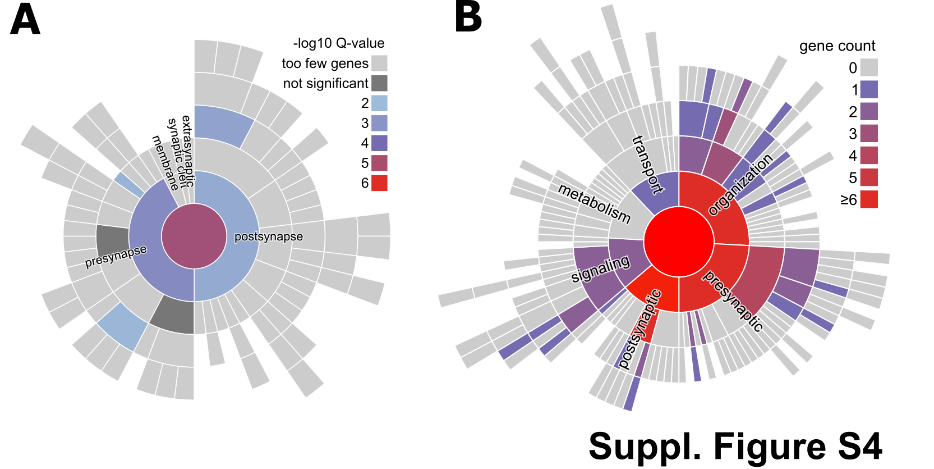


**Suppl Figure S4. Analysis of protein ontology of BDEVs dUC-isolated from the supernatant under microfluidic condition.** (**A**) Associated protein localization of top 100 ranked proteins. (**B**) Associated protein function of top 100 ranked proteins.

**
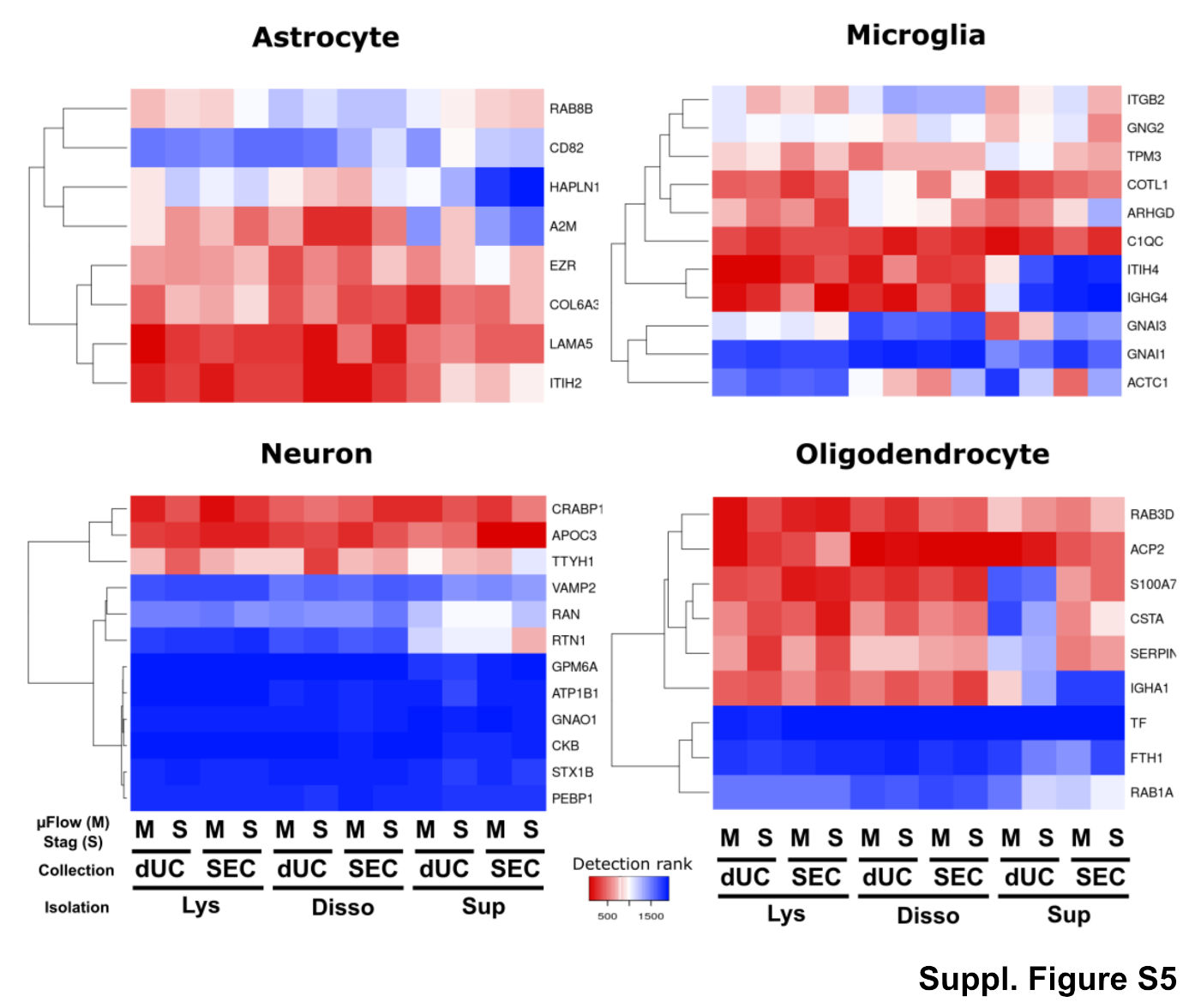
**

**Suppl Figure S5. OPAB-derived BDEVs have various cellular origin.** Heatmaps representing the rank assigned to indicated proteins in our proteomic analysis. The selected proteins represent specific cell type markers based on ^16^.

**
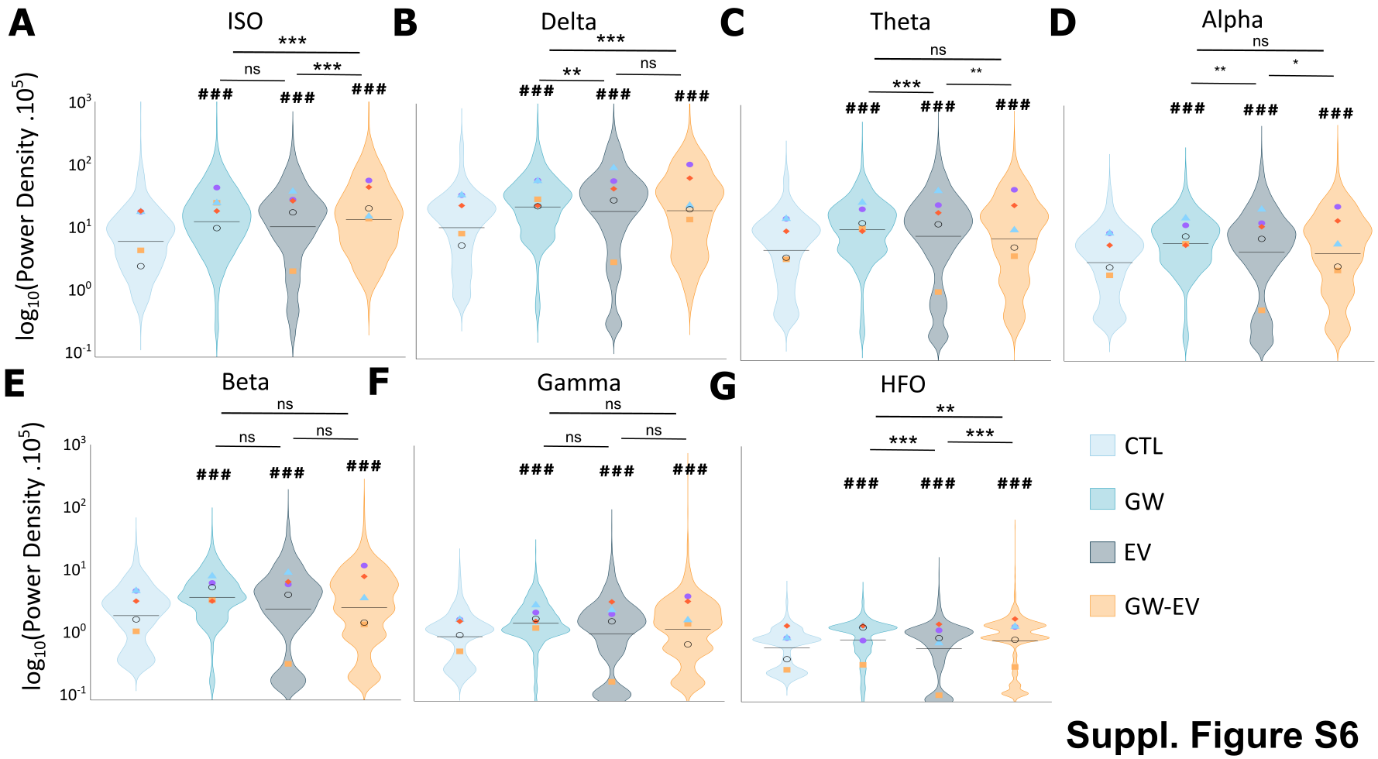
**

**Suppl Figure S6. Impact of BDEVs on LFP analysis at each frequency domain.** Power densities at each brain wave-associated frequency domains. Statistical analysis was performed using multiple t-test with Bonferonni correction, and post-hoc Tukey-HSD multiple comparisons, and p value < 0.05 (*), 0.01 (**), 0.005 (***), and 0.001 (****) between indicated conditions and p value < 0.005 (^###^) compared to CTL condition.

**Table S1.** Clinical information of the donors from which the brain explants were obtained.

**Table S2.** Proteomic dataset.

**Table S3.** Ontology terms associated to each condition.

**Table S4.** Shared most expressed proteins between conditions.
